# Impact of historical founder effects and a recent bottleneck on MHC variability in Commander Arctic foxes (*Vulpes lagopus*)

**DOI:** 10.1002/ece3.42

**Published:** 2012-01

**Authors:** Anna I Ploshnitsa, Mikhail E Goltsman, David W Macdonald, Lorna J Kennedy, Simone Sommer

**Affiliations:** 1 Department of Vertebrate Zoology, Lomonosov Moscow State University1/12 Leninskie gory, 119991 Moscow, Russia; 2 Evolutionary Genetics, Leibniz Institute for Zoo and Wildlife Research (IZW)D-10315 Berlin, Germany; 3 Wildlife Conservation Research Unit, Department of Zoology, University of Oxford, The Recanati-Kaplan CentreTubney House, Tubney, OX13 5QL Oxon, UK; 4 Centre for Integrated Genomic Medical Research, University of ManchesterM13 9PT Manchester, UK

**Keywords:** Bottleneck, Founder effect, Isolation, MHC Class II, *Vulpes lagopus*

## Abstract

Populations of Arctic foxes (*Vulpes lagopus*) have been isolated on two of the Commander Islands (Bering and Mednyi) from the circumpolar distributed mainland population since the Pleistocene. In 1970–1980, an epizootic outbreak of mange caused a severe population decline on Mednyi Island. Genes of the major histocompatibility complex (MHC) play a primary role in infectious disease resistance. The main objectives of our study were to compare contemporary variation of MHC class II in mainland and island Arctic foxes, and to document the effects of the isolation and the recent bottleneck on MHC polymorphism by analyzing samples from historical and contemporary Arctic foxes. In 184 individuals, we found 25 unique MHC class II DRB and DQB alleles, and identified evidence of balancing selection maintaining allelic lineages over time at both loci. Twenty different MHC alleles were observed in mainland foxes and eight in Bering Island foxes. The historical Mednyi population contained five alleles and all contemporary individuals were monomorphic at both DRB and DQB. Our data indicate that despite positive and diversifying selection leading to elevated rates of amino acid replacement in functionally important antigen-binding sites, below a certain population size, balancing selection may not be strong enough to maintain genetic diversity in functionally important genes. This may have important fitness consequences and might explain the high pathogen susceptibility in some island populations. This is the first study that compares MHC diversity before and after a bottleneck in a wild canid population using DNA from museum samples.

## Introduction

The major histocompatibility complex (MHC) is one of the most important genetic systems for infectious disease resistance in vertebrates (Klein 1986; Hedrick and Kim 2000). Characteristically, MHC genes show high allelic diversity, long allelic persistence times and high heterozygosity (Klein et al. 1993). The high levels of polymorphism of MHC genes is believed to be the result of balancing selection leading to the long-term maintenance of allelic lineages (trans-species polymorphism model) as well as positive/diversifying selection for amino acid replacement identified at the molecular level by an increased ratio of nonsynonymous over synonymous substitutions (*d*_N_*/d*_S_) at functionally important antigen-binding sites (ABS) (Hughes and Nei 1988, 1989). This diversity allows the binding of a variety of pathogen-derived antigens that initiate the appropriate immune response. A number of studies have underscored the influence of MHC diversity patterns on individual fitness and long-term persistence of populations and species (see reviews by Sommer 2005; Piertney and Oliver 2006, but see Radwan et al. 2010). The mechanisms responsible for maintaining polymorphism at MHC genes include negative frequency dependent selection (Snell 1968; Borghans et al. 2004) and heterozygote advantage (Doherty and Zinkernagel 1975), which are not mutually exclusive. Frequency dependence arises because the carriers of common alleles are more likely to be invaded by coevolving parasites while new and thus rather rare MHC alleles cause a temporary advantage (Trachtenberg et al. 2003). Heterozygosity allows presentation of a wider range of pathogen-derived peptides, and thus provides greater resistance to infection (Carrington 1999; Penn et al. 2002). Disassortative mating, mother–fetus interaction, and spatio-temporal variations in pathogen-mediated selective regimes also contribute to the maintenance of high MHC diversity (Hedrick 1999; Sommer 2005).

It is well known that isolation and large demographic declines affect the ability of a population to maintain genetic diversity over time due to increased action of genetic drift (Frankham 1997; Keller and Waller 2002). Genetic drift may overcome balancing selection, leading to reduced variation at MHC loci. A number of studies have documented severely reduced MHC variation in small, bottlenecked populations and endangered species (e.g., Ellegren et al. 1993; Radwan et al. 2007; Siddle et al. 2007). These species may be more susceptible to disease and thus more prone to extinction. Therefore, understanding the role of selection in maintaining MHC variation in bottlenecked populations has implications for the conservation of endangered species (O’Brien and Evermann 1988; Hughes 1991; Radwan et al. 2010).

**Figure 1:**
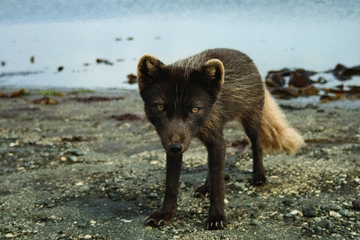
An adult Commander Arctic fox (*Vulpes lagopus*), Bering Island, 2009.

**Figure 2:**
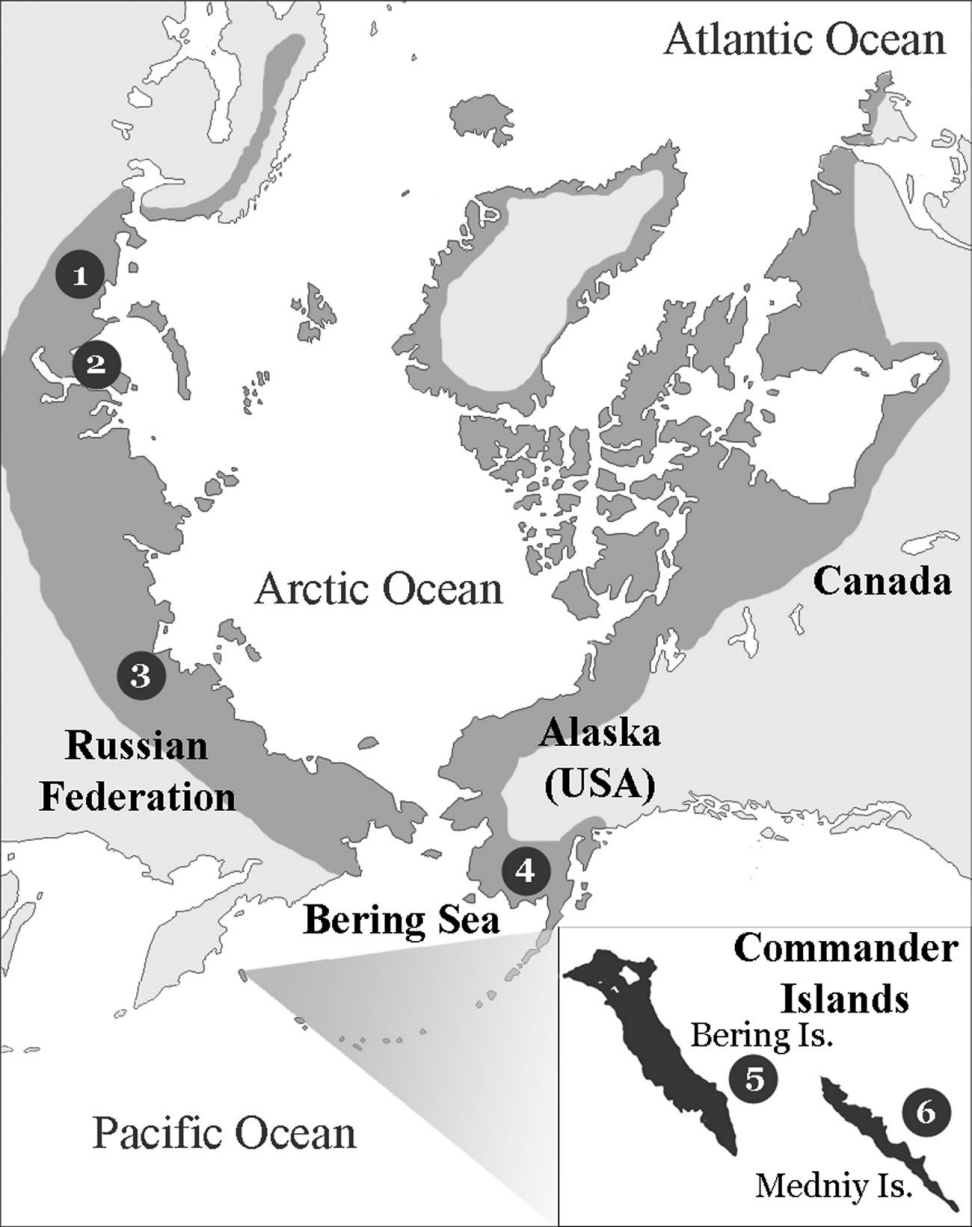
Geographic range of Arctic foxes and the sampling locations of continental (1 = Nenetsia, 2 = Yamal Peninsula, 3 = Lena River, 4 = Alaska) and Commander Island populations (5 = Bering Island, 6 = Mednyi Island).

The Arctic fox (*Vulpes lagopus*, previously *Alopex lagopus*) has a circumpolar distribution (Fig. 1). Most of the mainland populations (Eurasia and North America) have no apparent borders and show pronounced gene flow (Geffen et al. 2007; Norén et al. 2010). The Commander Islands (Russia) populations live at the southern edge of the species’ geographic range in the Pacific Ocean and have been isolated since the Pleistocene (Fig. 2). They are thought to represent two endemic subspecies (Bering Island: *V. l. beringensis*, Mednyi Island: *V. l. semenovi*). The islands were discovered in 1741, and at that time the Arctic fox was abundant on both islands. Subsequently, both populations were hunted heavily for their fur, and up to half of each island population was killed annually (Il’ina 1950). However, up to the middle of the 20th century, numbers remained relatively stable with about 2000–4000 foxes on Bering and up to 1000 on Mednyi (Geptner and Naumov 1967). In 1970–1980, the Mednyi population crashed due to epizootic mange (Goltsman et al. 1996). Nowadays, the population of Mednyi Arctic foxes is <100 individuals, 10–15% of its former abundance (Goltsman et al. 2005). Cases of the disease also occurred on Bering Island, but there it had no appreciable effect on the population size, which remained stable (at about 600 adult animals) (Ryazanov 2002).

Levels of neutral genetic variability in Commander Arctic foxes were analyzed in several independent studies and revealed reduced diversity compared to mainland populations (Dzhykiya et al. 2007; Geffen et al. 2007; A. I. Ploshnitsa, unpubl. data). A preliminary examination of MHC Class II DRB exon 2 in 12 contemporary Mednyi Arctic foxes showed monomorphism (Dzhykiya et al. 2008).

Museum specimens can reveal changes in populations’ genetic composition over time (Taylor et al. 1994). Usually, studies of the historical genetic variability in wild populations employ neutral markers such as mtDNA D-loop and/or microsatellites (Weber et al. 2004; Nyström et al. 2006). However, variation at neutral loci cannot detect selective processes involving the interaction of individuals with their environment or on the capacity for future adaptive changes (Meyers and Bull 2002; van Tienderen et al. 2002; Sommer 2005).

Our objectives were to (1) investigate the diversity of two MHC class II genes (DQB, DRB), the phylogenetic relationships between alleles, and evidence for selection processes on the molecular level, (2) compare contemporary variation of the MHC class II in continuous mainland (Alaska and Siberia) and insular Commander (Bering and Mednyi Islands, North Pacific) Arctic foxes, and (3) document the effects of the recent bottleneck on the functionally important MHC polymorphism in Mednyi Island Arctic foxes by comparing museum samples collected before the population crash with contemporary Arctic foxes.

## Materials and methods

### Sampling and DNA extraction

The genetic diversity of the ß-chain of MHC class II DRB and DQB genes was investigated in 47 mainland and 137 Arctic foxes from the Commander Islands. The mainland samples included Arctic foxes from the Russian Siberia population (Nenetsia, *n* = 2; Yamal Peninsula, *n* = 27; Lena River, *n* = 4) and 14 Arctic foxes from the North Shore of Alaska (USA) (Fig. 2). The Siberian and Alaskan samples were pooled as no segregation has been found between continental Arctic foxes. Survey of mtDNA and microsatellites did not indicate population differentiation between Arctic foxes connected by land or pack ice (Dalén et al. 2005; Geffen et al. 2007). Island Arctic foxes were represented by museum samples (collected in 1911–1946; stored at the Zoology Museum of Lomonosov Moscow State University) and contemporary samples (collected in 1997–2009). Fifty-two Mednyi and 15 Bering museum samples were tested, but results were obtained for only 43 and nine samples, respectively. Contemporary samples included 41 from Mednyi and 44 from Bering. Museum samples comprised mandibular bones with teeth, while contemporary samples were ear biopsies, skin or muscle tissues, stored in 96% ethanol.

DNA was extracted from contemporary samples using either phenol–chloroform (Sambrook et al. 1989) or DNeasy kit (Qiagen). DNA was extracted from museum samples from fine powder obtained from bones and teeth following a protocol by Yang et al. (1998) with modification using the QIAquick PCR purification kit (Qiagen). Museum samples were transferred to a room dedicated to ancient DNA extraction. Each bone was washed in water with a disposable brush, rinsed with deionized water, and sterilized by exposure to UV light for 60 min. The bone/tooth powder was obtained using stomatologic borers. Proteinase K (250 units, Qiagen) and 700-µl E buffer (EDTA = 0.5 M; Tris-HCl = 10 mM; SDS = 0.5%; pH = 8.5) were added to each powder sample and incubated for 24 h on a shaker at 55°C. After incubation, the samples were centrifuged at 2000 rpm for 5 min. The liquid fraction (ca. 600 µl) was transferred to a 5-mL tube and 3-mL PB buffer (Qiagen) was added. The solution was vortexed and passed through a column by centrifugation at 14,000 rpm for 1 min. After filtration, 720-µl PE buffer (Qiagen) were added to the column and centrifuged again (14,000 rpm, 1 min). DNA was dissolved in 40-µl EB buffer (Qiagen) and transferred to a new tube by centrifugation (14,000 rpm for 1 min, performed twice). DNA extracts from contemporary and museum samples were stored separately at –20°C.

### Contamination controls and authenticity of museum samples

The principal rules established for working with ancient DNA were followed (Cooper and Poinar 2001). Bone drilling, DNA extraction, and polymerase chain reaction (PCR) from museum samples took place in a separate room in a laminar box dedicated to ancient DNA research, which had never been used for contemporary Arctic fox DNA. In order to avoid cross-contamination between samples, only one sample at a time was drilled. Before each drilling, work surfaces and equipment were washed with chlorine solutions and/or UV irradiated. Each amplification contained at least two negative controls, which were continually screened and no evidence of contamination was observed. For 10 museum Arctic foxes, the DNA extraction was repeated and the derived sequences were compared to those obtained from the first extraction. The analysis from the same specimens produced identical results.

### PCR amplification of MHC Class II genes

Primers designed to amplify DRB (DM-1 5′-AAGTCCGAGTGCTATTTCACC-3′/DM-2 5′-TCGCCGCTGCACCGTGAAGCT-3′, Hedrick et al. 2000) and DQB (DQB-F 5′-CATGTGCTACTTCACCAACGG-3′/DQB-R 5′-CTGGTAGTTGTGTCTGCACAC-3′, Fernandez–Vina and Bignon 1997) were used. These primers gave products of 207 bp and 172 bp, respectively. The DRB primers (DM-1/DM-2) were specifically developed for the canid DRB locus and have been used for several species (*Canis lupus*, *C. l. familiaris*, *C. rufus*, *C. latrans*; Hedrick et al. 2000). The DQB-F/DQB-R primers (Fernandez–Vina and Bignon 1997) were originally designed as generic DQB primers based on several different species. Amplifications were performed in 20-µl volumes containing 1–5 µl of DNA extract, 0.2 mМ dNTP's (Q BIOgene), 1× PCR-buffer without detergents (Qiagen), 4 U Hotstar *Taq* polymerase (Qiagen), 0.5 µM each primer, 2 mМ MgCl_2_ (Q BIOgene), and sterile deionized water. PCR was performed using a T-Gradient Thermocycler 96 (Biometra) or MJ research PTC-200 (Bio-Rad). The initial activation at 95°C for 15 min was followed by 35–45 cycles consisting of 60-sec denaturation at 94°C, 60-sec annealing at 55°C (for DQB-F/DQB-R) or 59°C (for DM-1/DM-2), and 60-sec extension at 72°C, with a final 10-min extension at 72°C. For verification of successful amplification and absence of contamination, PCR-products were visualized in ethidium-bromide stained 2.0% agarose gels.

### Single-stranded conformation polymorphism (SSCP)

PCR products were subjected to SSCP analysis. A total of 2–5 µl of PCR product was mixed with 8–10 µl denaturing loading dye (prepared after ETC-manual, ETC Elektrophoresetechnik), denatured for 10 min at 90°C, and immediately chilled on ice before loading 6 µl on the SSCP gel. Fifteen percent polyacrylamide gels (CleanGel DNA-HP, ETC Elektrophoresetechnik) were prepared following the manufacturer's manual and run on a horizontal cooling electrophoresis system (Amersham Pharmacia Biotech). Temperature, power, and acrylamide concentration affected the running time and had to be optimized. Maximum separation was reached at constant conditions: 200 V, 20 mA, 10 W for 20 min followed by 450 V, 30 mA, 20 W for 4 h at 12°C. After separation, the gels were fixed and silver-stained (DNA Plus One Silver Staining Kit, Amersham Pharmacia Biotech). Samples were analyzed at least twice. In addition to new samples, all known alleles were run as reference on each SSCP gel. At least three examples from each SSCP band were cut separately from the gel, eluted in 1× TBE (EDTA = 2mM; Tris Base = 89 mM; boric acid = 89 mM; pH = 8.0) and reamplified by PCR using the primers described above prior to sequencing.

### Sequencing

The PCR products (1–5 µl) were purified using 2 U of Exonuclease I and 5 U of calf alkaline phosphotase (Fermentas). The mixture was incubated at 37°С for 15 min and heated to 85°С for 15 min. Sequencing of each PCR product was performed at least twice bidirectionally using a dye terminator sequencing kit BigDye® Terminator v 1.1 or v 3.1 Cycle Sequencing Kit with an Applied Biosystems® Automated Sequencer model 3100 following the manufacturer's instructions. Homozygous individuals were sequenced directly from the initial PCR product to confirm that a second allele was not present. Special care was given to all museum DNA templates and all analyses were repeated at least three times using independent set ups (PCR, sequencing, etc.). To exclude allele dropout for museum samples, direct sequences were completed for heterozygotes too. Sequences obtained from direct sequencing were compared to those obtained after cutting and reamplification of the respective SSCP bands and revealed similar results.

### Data analysis

MHC nucleotide sequences were edited, aligned, and translated into the corresponding amino acid sequences using the MEGA version 3.1 software packages (Kumar et al. 2001). The software package Arlequin, version 3.1 (Excoffier et al. 2006) was used to estimate nucleotide and amino acid diversity, observed (*H*_o_) and expected (*H*_e_) heterozygosities. Amino acid positions involved in antigen binding were identified by comparison with the antigen-binding groove structure of the human class II molecule (Brown et al. 1988, 1993). Relative frequencies of nonsynonymous (*d*_N_) and synonymous (*d*_S_) substitutions were calculated in MEGA 3.1 for the ABS and non-ABS according to Nei and Gojobori (1986), applying the correction of Jukes and Cantor (1969) for multiple hits. The *d*_N_/*d*_S_ ratios were tested for significant differences from neutrality with a *Z*-test (Tajima 1989; Nei and Kumar 2000). A Tatjima D test was performed using the MEGA version 5.0 (Tamura et al. 2011). NETWORK 4.5.1.0 (Bandelt et al. 1999) was used to construct median-joining networks. MEGA 3.1 was employed to construct a phylogenetic tree using the neighbor-joining algorithm (Saitou and Nei 1987) and Jukes–Cantor model. The stability of the inferred topology was assessed via bootstrap analysis with 1000 iterations. The extent of population differentiation at MHC DRB and DQB genes was examined by pairwise *F*_ST_ (10,000 permutations, Wright 1965) and the Global test (Markov chain length: 10,000 steps) using Arlequin version 3.1. Bonferroni corrected significance levels were applied for multiple comparisons (Rice 1989, Sachs 1992). FSTAT version 2.9.3 (Goudet 1995) program was used to calculate allelic richness based on the minimum sample size. Variants of MHC genes are referred to as alleles. Combinations of MHC alleles that are inherited together are referred to as haplotypes. There is extremely high linkage disequilibrium between MHC loci in all mammals, and this can be exploited to assign haplotypes. Thus, two locus (DRB, DQB) haplotypes were established using an interactive and subtractive approach as described previously for assigning dog haplotypes (Kennedy et al. 2002a, b). First, all foxes that were homozygous at both loci were selected, and from these several different DRB/DQB haplotype combinations were identified. These haplotypes were also found in heterozygous foxes, and by subtraction, the other haplotype in those foxes could be detected. In many cases, the subtractions revealed haplotypes that had already been identified in homozygous foxes. Second, the remaining foxes were examined using the haplotype data already identified, with further possible haplotypes assigned. There is a theoretical potential for misassignment of haplotypes using this method, but in practice it is easy to assign them, and there were no foxes for which we were uncertain of the haplotype assignment.

## Results

### Diversity and phylogenetic analyses of MHC class II Vula-DRB and Vula-DQB alleles

In total, 184 Arctic foxes were successfully investigated for both DRB and DQB MHC class II genes. However, the primers designed specifically to amplify DRB and DQB loci in other species were not locus specific in Arctic foxes. SSCP runs and direct sequencing revealed that both sets of primers amplified alleles from both loci simultaneously. Thus, DM-1/DM-2 amplified all DRB alleles plus all DQB alleles except *Vula*-DQB*01, *08, and *12, while DQB-F/DQB-R amplified all *Vula*-DQB alleles plus *Vula*-DRB*04 and *05. Thus the three alleles that were only amplified by the DQB primers had slightly shorter sequences than the other 22 alleles.

**Figure 3:**
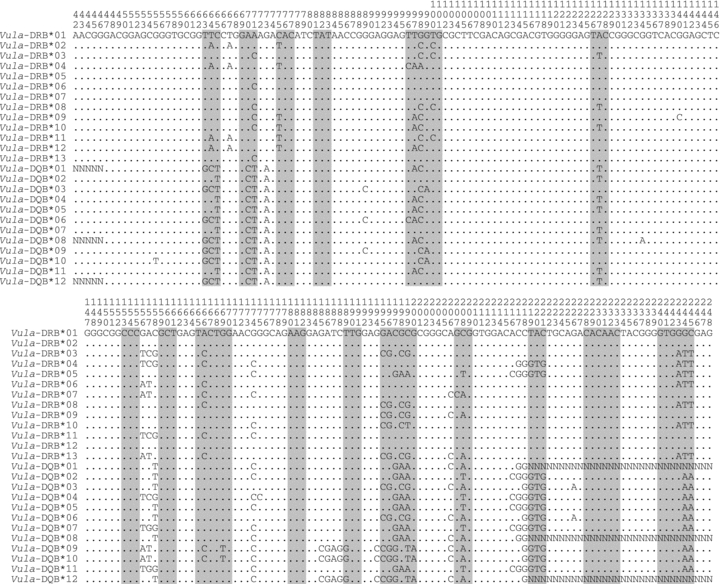
Nucleotide sequence variation of MHC class II *Vula*-DRB and *Vula*-DQB genes (exon 2) encoding the antigen-binding sites (ABS) in Arctic foxes (*V. lagopus*). Dots mark identity with the top sequence. ABS location is shadowed in gray according to Brown et al. (1988, 1993).

**Figure 4:**
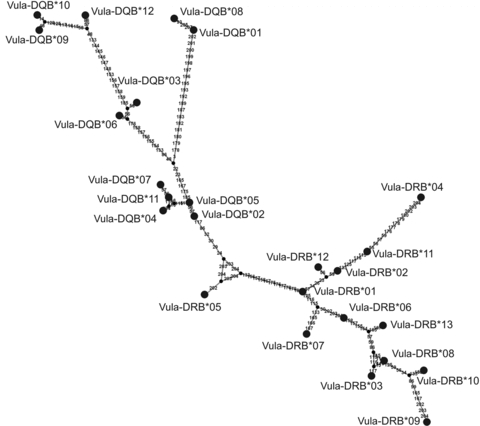
A median network of MHC class II DQB and DRB sequences found in Arctic foxes. Missing alleles (or latent vertices) are marked with small size dots. The distance between the alleles corresponds to the number of substitutions.

**Figure 5:**
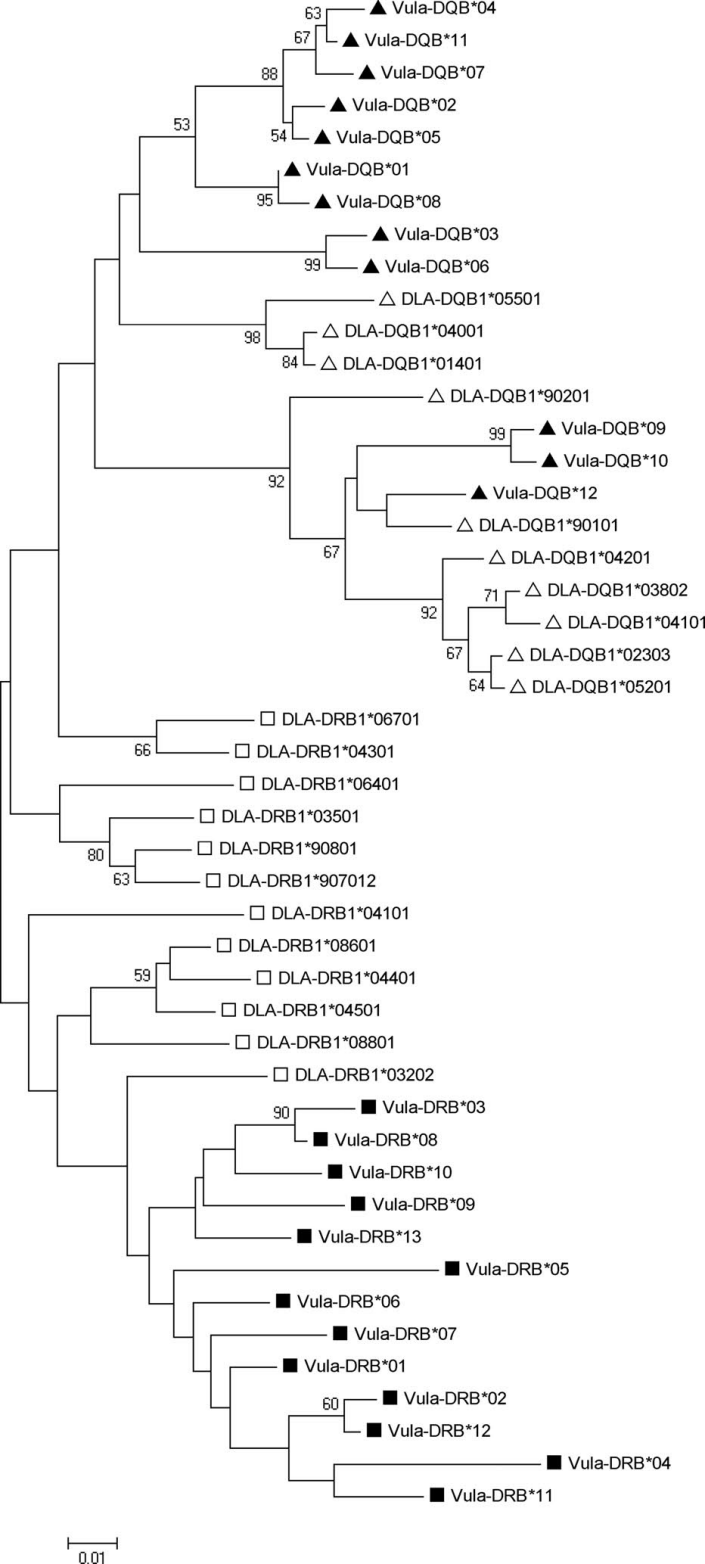
A neighbor-joining tree of the MHC class II sequences (*Vula*-DRB*01–*13 and *Vula*-DQB*01–*12) found in Arctic foxes analyzed together with other canid DRB and DQB alleles available in Genbank. The bootstrap values are indicated at the nodes of the branches. Empty squares = DRB alleles from other canid species, shadowed squares = DRB alleles found in Arctic foxes, empty triangles = DQB alleles from other canid species, shadowed triangles = DQB alleles found in Arctic foxes.

In total, 24 new and one previously published allele (*Vula*-DRB*01, Dzhykiya et al. 2008) were identified (Fig. 3). Nucleotide sequences are available at GenBank (EF690694 [Dzhykiya et al. 2008], HQ602687-HQ602710). Using phylogenetic analyses, the alleles were assigned to putative loci and labeled as *Vula*-DRB*01–*13 and *Vula*-DQB*01–*12 (Fig. 3) following the suggestion of Klein et al. (1990). The assignment to putative loci was derived from a median network (Fig. 4) and a neighbor-joining tree (Fig. 5). The median network showed that the alleles divided into two branches; *Vula*-DRB or *Vula*-DQB. The neighbor-joining tree confirmed the two main clusters and the assignment to *Vula*-DRB and *Vula*-DQB. *Vula*-DRB alleles clustered with DRB alleles from other canid species and *Vula*-DQB alleles clustered with other DQB canid sequences.

Thirty-seven different haplotypes were identified in Arctic foxes. The most common haplotypes were *Vula*-DRB*01/DQB*01 and *Vula*-DRB*02/DQB*02, (40.2% and 14.7%, respectively, see Table 1). The number of different MHC class II alleles within an individual ranged from two to five. The majority (91.3%) had one or two alleles at each locus. Three individuals (1.1%) had three *Vula*-DRB alleles and 13 individuals (7.6%) had three *Vula*-DQB alleles indicating that some haplotypes carried duplicated genes. The duplication of either *Vula*-DRB or *Vula*-DQB occurred for particular combinations of alleles. For example, alleles *Vula*-DRB*03 and *Vula*-DQB*12 were found only in haplotypes with three alleles. Therefore, these duplications may reflect ancient events.

**Table 1:** Frequency of MHC Class II haplotypes in Arctic foxes

				All			Alaska			Siberia			Historical Bering			Contemporary Bering			Historical Mednyi			Contemporary Mednyi		
										
DRB	DQB	DQB	DRB	Hom	Tot	%	Hom	Tot	%	Hom	Tot	%	Hom	Tot	%	Hom	Tot	%	Hom	Tot	%	Hom	Tot	%
*Vula*-DRB*01	*Vula*-DQB*01			65	148	40.2											7	8.0	24	59	68.6	41	82	100
*Vula*-DRB*02	*Vula*-DQB*02			9	54	14.7	3	15	53.6	1	11	16.7	1	5	27.8	4	23	26.1						
*Vula*-DRB*02	*Vula*-DQB*01			2	18	4.9				2	15	22.7					3	3.4						
*Vula*-DRB*04	*Vula*-DQB*02			1	15	4.1								4	22.2	1	11	12.5						
*Vula*-DRB*01	*Vula*-DQB*03				19	5.2								4	22.2		15	17.0						
*Vula*-DRB*05	*Vula*-DQB*01			3	18	4.9								1	5.6	2	9	10.2	1	8	9.3			
*Vula*-DRB*05	*Vula*-DQB*02			1	10	2.7										1	6	6.8		4	4.7			
*Vula*-DRB*05	*Vula*-DQB*02		*Vula*-DRB*03		2	0.5											2	2.3						
*Vula*-DRB*05	*Vula*-DQB*03	*Vula*-DQB*02			17	4.6								1	5.6		5	5.6		11	12.8			
*Vula*-DRB*05	*Vula*-DQB*03				8	2.2								2	11.1		3	3.4		3	3.5			
*Vula*-DRB*02	*Vula*-DQB*03				2	0.5								1	5.6		1	1.1						
*Vula*-DRB*01	*Vula*-DQB*02				4	1.1											3	3.4		1	1.2			
*Vula*-DRB*06	*Vula*-DQB*02				2	0.5		2	7.1															
*Vula*-DRB*07	*Vula*-DQB*01				8	2.2		2	7.1		6	9.1												
*Vula*-DRB*07	*Vula*-DQB*02				8	2.2		1	3.6		7	10.6												
*Vula*-DRB*07	*Vula*-DQB*06				3	0.8		2	7.1		1	1.5												
*Vula*-DRB*02	*Vula*-DQB*07				2	0.5		1	3.6		1	1.5												
*Vula*-DRB*12	*Vula*-DQB*02				4	1.1					4	6.1												
*Vula*-DRB*09	*Vula*-DQB*04				2	0.5					2	3.0												
*Vula*-DRB*09	*Vula*-DQB*04	*Vula*-DQB*12			3	0.8					3	4.5												
*Vula*-DRB*13	*Vula*-DQB*01				2	0.5					2	3.0												
*Vula*-DRB*09	*Vula*-DQB*09				2	0.5					2	3.0												
*Vula*-DRB*07	*Vula*-DQB*10				2	0.5					2	3.0												
*Vula*-DRB*09	*Vula*-DQB*11				2	0.5					2	3.0												
Other single haplotypes					13	5.2		5	18.0		8	12.0												
				81	368	100	3	28	100	3	66	100	1	18	100	8	88	100	25	86	100	41	82	100

Hom = number of animals homozygous for this haplotype, Tot = total number of animals with the haplotype, % = percentage with this haplotype.

**Figure 6:**
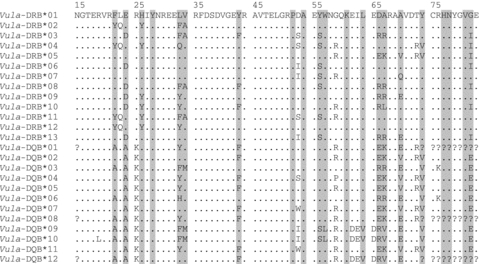
Amino acid variation of MHC class II *Vula*-DRB and *Vula*-DQB genes (exon 2) encoding the ABS in Arctic foxes (*V. lagopus*). Dots mark identity with the top sequence. The ABS amino acid positions are shadowed in gray (after Brown et al. 1988, 1993).

The number of variable nucleotides was 32 (16 amino acids) for *Vula*-DRB, and 34 (18 amino acids) for *Vula*-DQB (Figs. 3 and 6). The mean number of pairwise nucleotide differences in *Vula*-DRB alleles was 13.6 ± 2.3 (range: 2–25). *Vula*-DQB alleles differed on average by 14.6 ± 2.3 (range: 1–27) nucleotide positions. No insertions/deletions or stop codons were detected. All alleles had a unique amino acid sequence, except for *Vula*-DQB*01 and *Vula*-DQB*08, which differed by a single nucleotide site and translated into the same amino acid sequence. The number of amino acid differences between *Vula*-DRB alleles was 8.2 ± 1.9 (range: 2–13), and for *Vula*-DQB 8.1 ± 1.8 (range: 0–17).

### Evidence for selection processes on MHC class II in Arctic foxes

An alignment of amino acid sequences revealed that most variable positions were within the ABS (Fig. 6). Twelve of the 21 (57.1%) sites predicted to be involved in antigen recognition (Brown et al. 1988, 1993) were variable for *Vula*-DRB and 10 (47.6%) for *Vula*-DQB, whereas only four (8.3%) and eight (16.7%) of 48 non-ABS were polymorphic for *Vula*-DRB and *Vula*-DQB loci, respectively. The rates of synonymous (*d*_S_) and nonsynonymous (*d*_N_) substitutions were calculated separately for ABS and non-ABS (Table 2). For both loci, *d*_N_ was significantly higher than *d*_S_. Thus, the *d*_N_/*d*_S_ ratios were 3.3 (*P* = 0.009) and 3.4 (*P* = 0.007), for *Vula*-DRB and *Vula*-DQB, respectively. In contrast, for the non-ABS, the ratio between nonsynonymous (*d*_N_) and synonymous (*d_S_*) substitutions was not statistically significant (*d*_N_/*d*_S_ = 0.9). Moreover, *d*_N_ was 8.0 and 3.7 times higher in the ABS than in the non-ABS for *Vula*-DRB and *Vula*-DQB genes, respectively. Such pattern of substitutions provides evidence for positive, diversifying selection on the ABS leading to high amino acid polymorphism. The calculated positive Tajima's D signified low levels of both low- and high-frequency polymorphisms, which is a sign of a decrease in population size and/or balancing selection (Tajima 1989; Nei and Kumar 2000).

**Table 2:** Rates of nonsynonymous (*d*_N_) and synonymous (*d*_S_) substitutions (± standard error, SE) in the antigen-binding sites (ABS) and nonantigen-binding sites (non-ABS) as well as the resulting ratio *d*_N_*/d*_S_

Position	*N*	*d*_N_± SE		*d*_S_± SE		*d*_N_/*d*_S_	*P*-value
*Vula*-DRB							
ABS	21	0.22	0.06	0.07	0.05	3.3	0.009
Non-ABS	48	0.03	0.02	0.03	0.02	0.9	0.880
All	69	0.08	0.02	0.04	0.02	2.1	0.029
*Vula*-DQB							
ABS	21	0.19	0.06	0.06	0.04	3.4	0.007
Non-ABS	48	0.05	0.02	0.06	0.03	0.9	0.809
All	69	0.09	0.02	0.06	0.02	1.6	0.216

*N* = number of codons.

*P* = significance value assuming neutrality (*d*_N_ = *d*_S_) by using a *Z*-test.

### MHC class II diversity in mainland and insular contemporary and museum Arctic fox samples

Twenty MHC alleles were found in contemporary continental Arctic foxes: nine *Vula*-DRB and 11 *Vula*-DQB (Table 3). Despite the considerably larger sample size, only eight alleles were recorded in contemporary Commander Island foxes. Three alleles (*Vula*-DRB*02, *Vula*-DQB*01, and *02) were shared between both mainland and Commander Island populations (Table 3). Twenty-seven different haplotypes were found in continental Arctic foxes while only 12 haplotypes were found in Commander Arctic foxes (Table 1). The average gene diversity in contemporary continental Arctic foxes (0.707 ± 0.487 for Siberia and 0.568 ± 0.422 for Alaska) was similar to the average gene diversity in the contemporary Bering population (0.678 ± 0.469) and considerably higher than in the contemporary Mednyi population (0.000 ± 0.000). In the continental Arctic foxes, the observed and expected heterozygosity did not deviate from Hardy–Weinberg expectations but a significant deviation was observed in contemporary Bering Arctic foxes (Table 3).

**Table 3:** The relative frequency of MHC class II *Vula*-DRB and *Vula*-DQB alleles in Arctic foxes. For calculations of the average gene diversity over loci, observed heterozygosity (*H*_O_), expected heterozygosity (*H*_E_), Hardy–Weinberg *P*-value, and allelic richness (*R*_S_). Individuals with more than two DRB or DQB alleles were excluded (see Table 1)

	Contemporary Mainland			Commander Islands		
				
Alleles	Siberia *N* = 33	Alaska *N* = 14	Historical Bering *N* = 09	Historical Mednyi *N* = 43	Contemporary Bering *N* = 44	Contemporary Mednyi *N* = 41
*Vula*-DRB*01	–	–	0.44(4)	0.81(35)	0.50(22)	1.00(41)
*Vula*-DRB*02	0.73(24)	0.93(13)	0.56(5)	–	0.52(23)	–
*Vula*-DRB*03	–	–	0.11(1)	–	0.07(3)	–
*Vula*-DRB*04	–	–	0.33(3)	–	0.23(10)	–
*Vula*-DRB*05	–	–	0.44(4)	0.42(18)	0.45(20)	–
*Vula*-DRB*06	–	0.21(3)	–	–	–	–
*Vula*-DRB*07	0.39(13)	0.36(5)	–	–	–	–
*Vula*-DRB*08^1^	0.03(1)	–	–	–	–	–
*Vula*-DRB*09	0.27(9)	0.07(1)	–	–	–	–
*Vula*-DRB*10^1^	0.03(1)	–	–	–	–	–
*Vula*-DRB*11^1^	0.03(1)	0.07(1)	–	–	–	–
*Vula*-DRB*12	0.12(4)	–	–	–	–	–
*Vula*-DRB*13	0.09(3)	–	–	–	–	–
*H*_o_	0.69	0.64	0.89	0.11	0.73	Monomorphic
*H*_e_	0.71	0.56	0.80	0.33	0.75	
*P*	0.841	1.000	0.402	0.001	0.812	
*R*_s_	6.15	5.00	5.00	2.00	4.34	1.00
*Vula*-DQB*01	0.61(20)	0.29(4)	0.11(1)	0.95(41)	0.34(15)	1.00(41)
*Vula*-DQB*02	0.64(21)	0.93(13)	0.78(7)	0.33(14)	0.84(37)	-
*Vula*-DQB*03	–	–	0.67(6)	0.33(14)	0.52(23)	–
*Vula*-DQB*04	0.18(6)	–	–	–	–	–
*Vula*-DQB*05^1^	–	0.07(1)	–	–	–	–
*Vula*-DQB*06	0.06(2)	0.14(2)	–	–	–	–
*Vula*-DQB*07	0.03(1)	0.14(2)	–	–	–	–
*Vula*-DQB*08^1^	–	007(1)	–	–	–	–
*Vula*-DQB*09	0.12(4)	–	–	–	–	–
*Vula*-DQB*10^1^	0.06(2)	–	–	–	–	–
*Vula*-DQB*11^1^	0.06(2)	–	–	–	–	–
*Vula*-DQB*12	0.12(4)	–	–	–	–	–
*H*_o_	0.73	0.64	0.56	0.24	0.68	
*H*_e_	0.70	0.57	0.58	0.27	0.61	Monomorphic
*P*	0.256	0.620	1.001	0.122	0.004	
*R*_s_	6.66	6.00	3.00	2.88	3.00	1.00
Gene diversity	0.707 ± 0.487	0.568 ± 0.422	0.693 ± 0.498	0.299 ± 0.268	0.678 ± 0.469	0.000 ± 0.000

*N* = overall sample sizes.

1 These alleles were only found in one or two heterozygous animals.

In the contemporary, as well as in the historical Bering population, all eight alleles were present (Table 3). No significant shift in allele frequencies (*F*_ST_ = –0.009, *P* = 0.546) was observed in the Bering population over time (Table 4). *Vula*-DRB*02–*04 were unique to the Bering Island population, while the other five alleles were also observed in the museum samples of Mednyi Arctic foxes (Table 3). Thus, the Mednyi museum samples carried a subset of the alleles observed on Bering Island. In museum and contemporary Bering Arctic foxes, the average gene diversity was similar (0.693 and 0.678, respectively). A deficit in expected heterozygosity was observed in the *Vula*-DQB locus in contemporary Bering Arctic foxes (*H*_o_ = 0.68, *H*_e_ = 0.61, *P* = 0.004), while in the historic Bering population, no deviation from Hardy–Weinberg expectations was detected (Table 3). The differentiation between the Commander populations increased during the last century (from *F*_ST_ = 0.475 to 0.548, Table 4). A slight subdivision was found between continental Arctic foxes (*F*_ST_ = 0.052, *P* = 0.007). The highest differentiation was observed for contemporary Mednyi from either Bering or continental populations, while the lowest subdivision was registered between Bering population and contemporary continental Arctic foxes. The *F*_ST_ parameter calculated for *Vula*-DRB and *Vula*-DQB gene separately showed similar values. The results of the Global test also did not show population differentiation between historical and contemporary Bering Arctic foxes (*P* = 0.304).

**Table 4:** Pairwise *F*_ST_ values and *P*-value (upper triangle of the table) between Arctic fox populations calculated on the basis of MHC class II alleles

	Contemporary Siberia	Contemporary Alaska	Historical Bering	Historical Mednyi	Contemporary Bering	Contemporary Mednyi
Contemporary Siberia	/	0.007	0.000	0.000	0.000	0.000
Contemporary Alaska	0.052	/	0.000	0.000	0.000	0.000
Historical Bering	0.141	0.139	/	0.000	0.546	0.000
Historical Mednyi	0.392	0.564	0.475	/	0.000	0.000
Contemporary Bering	0.116	0.118	–0.009	0.333	/	0.000
Contemporary Mednyi	0.606	0.821	0.815	0.169	0.548	/

All contemporary Arctic foxes from Mednyi were monomorphic and had the same genotype consisting of *Vula*-DRB*01/DQB*01. These alleles were also detected in the Mednyi museum samples along with *Vula*-DRB*05, *Vula*-DQB*02, and *03. The most common alleles in former times were *Vula*-DRB*01 and *Vula*-DQB*01, which were present in 81% and 95% of the individuals, respectively. The majority (24 samples, 56%) of the historical Mednyi Arctic foxes shared the same genotype as the contemporary ones (Table 3). Thus, no MHC variability was found in contemporary Mednyi Arctic foxes, while for the Mednyi museum foxes, average gene diversity was 0.299 (Table 3). A heterozygosity deficit in the *Vula*-DRB gene was shown in the historical Mednyi population (*H*_o_ = 0.11, *H*_e_ = 0.33, *P* = 0.001).

## Discussion

Our overall aim was to investigate the impact of historical founder effects and a recent bottleneck on MHC class II DQB and DRB variability in Commander Arctic foxes (*V. lagopus*). A total of 25 alleles were observed. Evidence of positive selection for amino acid diversity was identified by an increased *d*_N_*/d*_S_ ratio at functionally important ABS. In mainland Arctic foxes, a higher MHC variability (20 alleles) was recognized, while insular Arctic foxes shared only eight alleles (Mednyi population: five alleles, Bering Island: eight alleles). A recent bottleneck caused the final loss of MHC variability in both DRB and DQB loci on Mednyi Island, while the bigger Bering population has been able to preserve its MHC diversity.

### Similarity of MHC class II Vula-DRB and Vula-DQB loci in Arctic foxes

DRB and DQB loci play an integral role in pathogen recognition and have similar structures (Klein 1986). Similarity between some alleles from these genes has been previously reported for other canid species and might be the result of selection, convergence, or more likely intergenic recombination (Seddon and Ellegren 2002). Our analyses revealed that the primers designed to amplify DRB and DQB loci separately were not gene specific in Arctic foxes. This might explain why our phylogenetic analyses, although mostly in agreement with current assignments with respect to loci of previous GenBank submissions, differ in some alleles. For example, the BLASTn search of the alleles *Vula*-DRB*01–*13 but also of the alleles *Vula*-DQB*01 and *08 revealed a 92–96% identity to published MHC class II DRB1 sequences of the dog (*C. l. familiaris*, AY220510, DQ056278, FJ415066), the African wild dog (*L. pictus*, FJ648561, FJ648562, FJ648563, FJ648570), the coyote (*C. latrans*, AY009520, AY009528, AF516923), the Ethiopian wolf (*C. simensis*, AM182465), and the wolf (*C. lupus*, AM408904). *Vula*-DQB*02–*07 and *09–*12 had a 93–97% similarity to DQB sequences of the dog (*C. l. familiaris*, AF043160, AB236363), the wolf (*C. lupus*, AY126648, FM246843), and the African wild dog (*Lycaon pictus*, FJ648575).

We found signs that some individuals have haplotypes that carry two copies of either *Vula*-DRB and/or *Vula*-DQB. Duplication events within a species and even within populations have been previously reported in some other species (Miller and Lambert 2004; Baker et al. 2006; Axtner and Sommer 2007) as well as in the domestic dog for both MHC class II (Kennedy et al. 2007) and class I (L. J. Kennedy, unpubl. data). Gene duplication is another mechanism by which the diversity of the MHC is increased.

There were no cases of subbanding or reaction failure in Arctic foxes, which would indicate the presence of primer mismatches at targeted annealing sites and/or nonspecific primer binding in other genomic regions (Smith et al. 2002). This makes the occurrence of null alleles unlikely, although it cannot be ruled out. It might be possible that some samples classified as heterozygous with two alleles at a single locus, in fact have duplicated loci in a homozygous state.

### Comparison of MHC class II to neutral variability in mainland and insular populations of Arctic foxes

Levels of neutral genetic variability in Commander Arctic foxes were analyzed in several independent studies. The examination of the control region of the mitochondrial DNA (mtDNA, hypervariable region I [HVRI], 610 bp) in 30 contemporary Mednyi foxes revealed only one haplotype, while in 30 contemporary Bering individuals, seven haplotypes were observed (Dzhykiya et al. 2007; but see also Geffen et al. 2007). A microsatellite variability study using 11 loci revealed only 2.5 and 4.0 alleles per locus with observed heterozygosities of 0.19 and 0.50 in contemporary Mednyi (*N* = 17) and Bering Arctic (*N* = 17) foxes, respectively (Geffen et al. 2007). The study of five other microsatellites detected 1.6 and 4.4 alleles per locus with an observed heterozygosity of 0.29 and 0.65 in contemporary Mednyi (*N* = 27, different individuals as above) and Bering foxes (*N* = 12, different individuals as above), respectively (A. I. Ploshnitsa, unpubl. data). Analysis of both the mtDNA and microsatellite markers in Mednyi museum Arctic fox samples (1911–1946, *N* = 36) showed an already depleted neutral variability before the population crash: no mtDNA variability was found and 2.2 alleles per locus were detected in five microsatellites (*H*_O_ = 0.36, A. I. Ploshnitsa, unpubl. data). Thus, the neutral data are in agreement with the results from the adaptive marker and indicated a reduced diversity in island compared to mainland populations and that Mednyi Arctic foxes showed lower diversity than Bering ones even though a direct statistical comparison is difficult due to the different modes of evolution. The frequently observed phenomenon that an insular small population maintains lower genetic diversity than a larger mainland one (Frankham 1997; White and Searle 2007) has been generally described for neutral markers, but our data revealed that it might also occur in an adaptive marker under selection such as MHC genes. Genetic drift operating on small island populations would have the effect of reducing diversity and would also counteract the effects of balancing selection if allele frequencies are near to 0 or 1 (Allendorf and Luikart 2007). A reduced MHC diversity has been observed in island populations while mainland populations maintained higher variability in the Australian bush rat (*Rattus fuscipes greyii*) (Seddon and Baverstock 1999) and the black-footed rock-wallaby (*Petrogale lateralis lateralis*) (Mason et al. 2010). Depletion of MHC variability often occurs together with a reduction of neutral diversity (Alcaide et al. 2010; Miller et al. 2010). A study on island foxes (Aguilar et al. 2004) represents an exceptional case. Authors showed MHC variability in animals, which had lost any neutral diversity. Using computer simulation, Aguilar et al. concluded that the observed MHC variability could be a result of unprecedentedly strong selection that followed a very “narrow” (less then 10 individuals) bottleneck, which resulted in the loss of microsatellite variability. However, the MHC variability in this study included assessing microsatellites in the MHC region and we have found at least one of these microsatellites to be unstable (L. J. Kennedy, unpubl. data), which throws some doubt onto whether the MHC is as variable as suggested. We studied a panel of 20 microsatellites in the canine MHC region in over 400 dogs from many different breeds, and also in several multigeneration dog families (L. J. Kennedy, unpubl. data). We showed that the same MHC class II haplotype in any particular breed had a characteristic pattern of microsatellite alleles. These patterns could be used to assign the MHC class II haplotypes. However, there were several microsatellites that had to be excluded from these patterns, because they did not show consistency between dogs of the same breed with the same haplotypes, and furthermore, the alleles did not inherit as expected in families. Offspring would have alleles of different length from their parents. The markers chosen by Aguilar. include some of these unstable microsatellites.

As with the results obtained from the neutral markers in Commander Arctic foxes (Dzhykiya et al. 2007; Geffen et al. 2007; A. I. Ploshnitsa, unpubl. data), the MHC analysis also showed signs of a founder effect. Twenty MHC alleles were found in continental Arctic foxes while eight alleles were found in the Commander populations. The lower allele number on Commander Islands compared to the continental Arctic foxes could be a result of a founder effect that occurred when Commander Arctic foxes separated from the mainland during the Pleistocene colonization. Three alleles were found in both mainland and insular Commander Arctic foxes and indicate a long-term persistence of particular MHC alleles, at least since the Pleistocene age. The evidence that some MHC alleles can be maintained by balancing selection for several thousand years has also been observed in other species (Babik et al. 2009). As expected, contemporary continental Arctic foxes were significantly different from the contemporary Bering population but this differentiation was much lower than that between contemporary Bering and contemporary Mednyi (Table 4). The analysis of microsatellites showed similar results. Thus, *F*_ST_ between Bering and continental Arctic foxes was 0.19–0.21, between Mednyi and continental Arctic foxes 0.37–0.38, while between Commander populations it was 0.41 (Geffen et al. 2007). In the historical population of the larger Bering Island, a higher MHC diversity was observed than in the historical Mednyi Island population that contains a subset of the alleles observed on Bering. This suggests that Bering Island was colonized first and that Mednyi was subsequently colonized from Bering. This explanation is further supported by mtDNA analysis (Dzhykiya et al. 2007).

The genetic variability was further reduced after the recent bottleneck on the contemporary Mednyi population. While it caused the loss of only three of 11 alleles in five microsatellite loci with a decrease in the observed heterozygosity from 0.36 to 0.29 (A. I. Ploshnitsa, unpubl. data), the epizootic infection caused the population on Mednyi Island to become monomorphic for both MHC genes. Nowadays, all individuals are homozygous for MHC *Vula*-DRB and DQB loci and only the most frequent alleles of the former Mednyi population (*Vula*-DRB*01, DQB*01) have survived. No significant differentiation between the museum and contemporary Bering population was observed and there was no sign of a bottleneck effects on Bering Island. The same eight MHC alleles were found at the same frequencies. During a bottleneck, the main force that reduces genetic variability in neutral markers is genetic drift, which mostly affects low frequency alleles. The fact that the loss of MHC diversity is similar to neutral loci and that during the epizootic the most frequent alleles became fixed in both DRB and DQB genes convinces us that genetic drift overcame balancing selection and led to the lack of MHC diversity in the contemporary Mednyi Arctic foxes. However, there is an alternative scenario: If the Mednyi MHC variation has decayed more than neutral markers, then the observed MHC monomorphism might be a result of strong positive directional selection if the most common MHC alleles before the epizootic or certain MHC haplotypes conferred a selective advantage to the individuals carrying those alleles or allele combinations.

Our study indicates that effective population size is one of the primary factors in a population's ability to maintain genetic variability over time. Moreover, functionally important MHC genes might be threatened by reduction of population size even more than neutral loci. Recent computer simulation revealed that the selection on MHC variation in bottlenecked populations may still be pronounced but, unlike in large populations, balancing selection acting on parasite resistance in a small population can have the opposite effect and deplete MHC variation even faster than expected under drift (Ejsmond and Radwan 2009). Most species that have experienced an extreme population bottleneck showed a depletion of variation at MHC loci (Ellegren et al. 1993; Babik et al. 2005; Mainguy et al. 2007; Radwan et al. 2007).

### Adaptive genetic diversity and fitness

If a population loses adaptive genetic diversity, one might expect the loss of fitness due to increased susceptibility to infection (O’Brein and Evermann 1988; Hughes 1991; Hedrick 2001). Reduced MHC variation has been associated with higher susceptibility to infectious diseases (Paterson et al. 1998; Hedrick and Kim 2000; Lachish et al. 2007). However, several studies report limited MHC variation in species that have undergone population bottlenecks, but yet the populations survived and even increased in number (Ellegren et al. 1993; Babik et al. 2005).

In 1741, when Arctic foxes were first described, the number of Mednyi foxes was high and their fur valuable; nowadays, as contemporary Mednyi Arctic foxes are MHC monomorphic, they show high rates of parasite infection and poor fur quality (Goltsman et al. 2005; N. A. Bocharova, unpubl. data). Even after protection, and despite the absence of any apparent environmental limitations (Goltsman et al. 2005), Mednyi Arctic foxes have not recovered to their former level. The larger population on Bering Island is also exposed to the mange parasites, but perhaps their higher MHC diversity increases their resistance to mange (Goltsman et al. 1994–2010). In conclusion, island Arctic foxes illustrate monomorphism at functionally important *Vula*-DRB and DQB genes due to a demographic bottleneck and high susceptibility to mange disease, which currently limits the recovery process.
